# Endoscopic treatment of obesity: A protocol of updated systematic review with network meta-analysis of randomized controlled trials

**DOI:** 10.1371/journal.pone.0308410

**Published:** 2024-09-06

**Authors:** Eun Jeong Gong, Chang Seok Bang, Gwang Ho Baik

**Affiliations:** 1 Department of Internal Medicine, Hallym University College of Medicine, Chuncheon, Korea; 2 Institute for Liver and Digestive Diseases, Hallym University, Chuncheon, Korea; 3 Institute of New Frontier Research, Hallym University College of Medicine, Chuncheon, Korea; Kyung Hee University School of Medicine, REPUBLIC OF KOREA

## Abstract

**Background:**

Obesity, characterized by excessive fat accumulation, poses a significant public health challenge globally. Recent advancements in medical technology have heralded the emergence of endoscopic bariatric treatments (EBTs) as innovative alternatives to conventional obesity interventions. Despite previous systematic reviews and network meta-analyses, they also highlighted discrepancies in outcomes and efficacy among different EBTs. Here, we will update a systematic review and network meta-analysis of randomized controlled trials (RCTs) focusing on EBTs and presents a protocol for the reproducibility and transparency.

**Methods:**

The core protocol of this study was registered at PROSPERO database (CRD42024514249) on Jan 2024. Core databases including MEDLINE through PubMed, Embase, and Cochrane library will be searched relevant studies, and a systematic review with network meta-analysis will be performed. Two evaluators (EJ Gong and CS Bang) will independently screen the titles and abstracts following the eligibility criteria; (1) RCTs investigated the compared the efficacy of EBTs and controls; (2) studies published in English; and (3) studies in full-text format. We will exclude studies meeting the following criteria; (1) studies that did not report the treatment outcomes, such as percent excess weight loss or percent total body weight loss; (2) case reports and review articles; (3) ineligible research objects, for example, animals or children; and (4) insufficient data regarding treatment outcome. The primary outcomes will be the common efficacy metric found after systematic review of relevant studies, such as percent excess weight loss or percent total body weight loss with a follow-up of at least 6 months. Narrative (descriptive) synthesis is planned and quantitative synthesis will be used if the included studies are sufficiently homogenous. The quality of the identified studies will be assessed using the Cochrane Risk of Bias assessment tool version 2.0 (ROB 2.0). All the systematic review and network meta-analysis process will be undertaken keeping the principles of the Preferred Reporting Items for a Systematic Review and Meta-analysis for systematic review protocols (PRISMA-P) and PRISMA Extension Statement for Reporting of Systematic Reviews Incorporating Network Meta-analyses of Health Care Interventions (PRISMA-NMA).

**Discussion:**

This updated systematic review and network meta-analysis will provide information about comparative efficacy of various EBTs and this will help physicians in the decision-making process for the selection of treatment modalities in the clinical practice.

## Introduction

Obesity is a global epidemic that adversely impacts millions worldwide, transcending age, socioeconomic status, and geographic boundaries. This chronic condition, marked by excessive fat accumulation, significantly elevates the risk for numerous health issues, including type 2 diabetes, cardiovascular diseases, certain cancers, and a spectrum of metabolic disorders [1]. The etiology of obesity is complex and multifactorial, incorporating genetic predispositions, lifestyle choices, environmental factors, and psychological aspects. Traditional treatment modalities for obesity, ranging from dietary and lifestyle modifications to pharmacotherapy and surgical interventions, offer varied success rates and are often hindered by high relapse rates and patient non-compliance [1, 2]. While new strategies, including endoscopic treatments, are being explored for their potential to be less invasive and more effective, they also come with their own set of challenges and risks. The search for sustainable and effective obesity management remains a high priority in public health and medical research.

Recent advancements in medical technology have heralded the emergence of endoscopic bariatric treatments (EBTs) as innovative alternatives to conventional obesity interventions [3]. EBTs encompass a variety of procedures designed to reduce gastric volume, alter gastrointestinal anatomy, or both, thereby facilitating weight loss and improving obesity-related conditions [3]. Among these, intragastric balloon placement temporarily reduces stomach capacity, encouraging earlier satiety [4, 5]. Endoscopic sleeve gastroplasty (ESG) mimics the effects of surgical sleeve gastrectomy by reducing the stomach size through suturing, but without the need for incisions [6]. Aspiration therapy provides a novel approach by allowing the removal of a portion of ingested food from the stomach through a specially designed tube, thus reducing calorie absorption [3]. Another technique, the duodenal-jejunal bypass liner (DJBL), mimics the effects of bariatric surgery by preventing the absorption of nutrients in the early part of the intestines [7]. These methods represent a significant shift towards minimally invasive approaches, offering potential benefits in terms of safety, recovery time, and overall effectiveness.

This paper presents a protocol of updated systematic review and network meta-analysis of randomized controlled trials (RCTs) focusing on EBTs. Despite previous systematic reviews and network meta-analyses conducted in 2019 and 2020 [8, 9], which provided valuable insights, they also highlighted discrepancies in outcomes and efficacy among different EBTs (Table 1). These differences underscore the dynamic nature of the field and the importance of incorporating new evidence to reconcile these variations.

**Table 1:** Comparison of previous meta-analyses with current study.

	Jung SH et al. (2020) [9]	Khan Z et al. (2019) [8]	Current study
**Number of included studies**	22 randomized studies	12 randomized studies (4 randomized studies & 8 observational studies)	Not yet started
**Main outcome**	%EWL and %TBWL	%EWL and %TBWL	%EWL and %TBWL
**Literature searching strategy (database)**	MEDLINE, Embase, Cochrane Library, LILACS, SCOPUS, and CINAHL databases (~Feb 2020)	MEDLINE, Embase, Cochrane database, and Scopus (~Nov 2017)	MEDLINE, Embase, and Cochrane Library
**Quality assessment of included studies**	The Cochrane Risk of Bias assessment tool version 1.0	The Cochrane Risk of Bias assessment tool version 1.0	The Cochrane Risk of Bias assessment tool version 2.0
**Follow-up duration**	3–12 months (variable). Most enrolled studies were less than 6 months	At least 6 months	All durations
**Results**	All bariatric endoscopic procedures (fluid-filled balloon, POSE, and DJB), except for gas-filled balloon and botulinum toxin injection, showed superior short-term efficacy in terms of %TBWL or %EWL compared with lifestyle modification	During a follow-up of 6–12 months, both AA and ESG had excellent efficacy in achieving significant and sustained weight loss; however, ESG was found to be superior in terms of weight loss when compared with POSE.	Not yet measured
**Measuring heterogeneity and the reason of heterogeneity**	Cochrane’s Q test (*I*^*2*^ statistics). Heterogeneity was found, but no reason for heterogeneity is described.	Cochrane’s Q test (*I*^*2*^ statistics). Authors conducted predetermined subgroup analysis based on months of follow-up. Authors encountered considerable heterogeneity in few of our estimates and outlier study was found. But the %EWL at 12 months did not have any heterogeneity among the three procedures	If heterogeneity was found, we will conduct the sensitivity, subgroup, and meta-regression analysis according to the found modifiers to confirm the reason of heterogeneity. Publication bias will be assessed.

%EWL, percent excess weight loss; %TBWL, percent total body weight loss; ESG, endoscopic sleeve gastroplasty; AA, AspireAssist; POSE, primary obesity surgery endoluminal; DJBL, duodenal-jejunal bypass liner.

### Objectives

Primary objective. This study aimed to evaluate the comparative efficacy of EBTs for morbid obesity, addressing the gap left by earlier reviews and providing an updated perspective on the effectiveness of these interventions in light of the evolving evidence base.

Secondary objectives. If heterogeneity among enrolled studies were found, we will assess the reason of heterogeneity for the interpretation of the results of this systematic review.

## Materials and methods

### Review question

We used the patient/population, intervention, comparison, outcome (PICO) as a research question formation and search strategy tool [10].

### Population

Patients with morbid obesity (body mass index will be collected for each study).

### Intervention

EBTs, including intragastric balloon, DJBL, aspiration therapy, endoscopic gastric plication, primary obesity surgery endoluminal (POSE), and botulinum toxin injection, etc.

### Control

Sham and/or lifestyle modification.

### Outcome

The primary outcomes will be the common efficacy metric found after systematic review of relevant studies, such as percent excess weight loss (%EWL) or percent total body weight loss (%TBWL).

The final review question is that the comparative the efficacy of EBTs in the treatment of morbid obesity.

### Overall study design

This systematic review with network meta-analysis will be performed in accordance with the statement of the Preferred Reporting Items for a Systematic Review and Meta-analysis for systematic review protocols (PRISMA-P) and PRISMA Extension Statement for Reporting of Systematic Reviews Incorporating Network Meta-analyses of Health Care Interventions (PRISMA-NMA) [11–14]. The core protocol of this study was registered at PROSPERO database (CRD42024514249) on Jan 2024. Any amendment in protocol or methodology of this study will be described in the following publication.

### Eligibility criteria for the study selection

The publications included in this systematic review must meet the following inclusion and exclusion criteria; Studies will be included if they satisfied the following criteria: (1) randomized controlled studies investigated the compared the efficacy of endoscopic bariatric procedures and controls; (2) studies published in English; and (3) studies in full-text format.

The exclusion criteria is as follows: (1) studies that did not report the treatment outcomes, such as %EWL or %TBWL; (2) case reports and review articles; (3) ineligible research objects, for example, animals or children; and (4) insufficient data regarding treatment outcome.

When there are duplicate data reported from the same institution, the studies with the largest number of enrolled population which may encompass data from all studies will be included.

### Information sources (literature search) and study selection process

Firstly, two authors (C.S.B. and E.J.G) will independently perform a web-based core database search (MEDLINE-PubMed, Embase, and the Cochrane Library) using common search formulas (from inception to Feb 2024). Any duplicate articles will be excluded. The titles and abstracts of all identified articles will be reviewed, and irrelevant articles will be excluded. Full-text reviews will subsequently be carried out to determine whether the pre-established inclusion criteria are satisfied in the identified publications. References will be also reviewed to identify any additional relevant studies. Disagreements between the authors will be resolved by discussion or consultation with a third author (G.H.B.). References will be exported into the Endnote software (Clarivate, Berkeley, California, United States).

### Searching strategy

The search formulas were made using common keywords relevant to EBTs for the treatment of obesity (inception to Feb 2024). Medical Subjects Headings (MeSH) keywords for MEDLINE and Emtree keywords for Embase were included in the searching formula. The detailed search formulas to identify the relevant articles are described in Table 2.

**Table 2:** Searching formula to find the relevant articles.

**Database: MEDLINE (through PubMed)**
#1 "obesity"[tiab] OR "obesity"[Mesh] #2 "endoscopic bariatric therapy"[tiab] OR "bariatric endoscopy"[tiab] OR "intragastric balloon"[tiab] OR “gastric balloon”[tiab] OR "stomach balloon"[tiab] OR "stomach bubbles"[tiab] OR "Ballobes"[tiab] OR "BioEnterics "[tiab] OR "Reshape"[tiab] OR "BIB"[tiab] OR "Obalon"[tiab] OR "Obera"[tiab] OR "Heliosphere"[tiab] OR "Garren-Edwards"[tiab] OR "Taylor"[tiab] OR "Endobarrier"[tiab] OR "Liner"[tiab] OR "Duodeno-jenjunal bypass"[tiab] OR "Duodenojenjunal bypass"[tiab] OR "POSE"[tiab] OR "primary obesity surgery endoluminal"[tiab] OR "endoluminal surgery"[tiab] OR "endoscopic sleeve"[tiab] OR "endoscopic gastroplasty"[tiab] OR "endoscopic gastric plication"[tiab] OR "overstitch"[tiab] OR "Apollo"[tiab] OR "AspireAssist"[tiab] OR "aspiration therapy"[tiab] OR "gastrostomy tube"[tiab] OR "botulinum"[tiab] OR "botox"[tiab] OR "BTX-A"[tiab] OR "gastrostomy tube"[tiab] #3 #1 AND #2 #4 #3 AND English[Lang] #5 #4: Filters: Randomized Controlled Trial:
**Database: Embase**
#1 ’obesity’:ab,ti,kw #2 ’endoscopic bariatric therapy’:ab,ti,kw OR ’bariatric endoscopy’:ab,ti,kw OR ’intragastric balloon’:ab,ti,kw OR ’gastric balloon’:ab,ti,kw OR ’stomach balloon’:ab,ti,kw OR ’stomach bubbles’:ab,ti,kw OR ’Ballobes’:ab,ti,kw OR ’BioEnterics’:ab,ti,kw OR ’Reshape’:ab,ti,kw OR ’BIB’:ab,ti,kw OR ’Obalon’:ab,ti,kw OR ’Obera’:ab,ti,kw OR ’Heliosphere’:ab,ti,kw OR ’Garren-Edwards’:ab,ti,kw OR ’Taylor’:ab,ti,kw OR ’Endobarrier’:ab,ti,kw OR ’Liner’:ab,ti,kw OR ’Duodeno-jenjunal bypass’:ab,ti,kw OR ’Duodenojenjunal bypass’:ab,ti,kw OR ’POSE’:ab,ti,kw OR ’primary obesity surgery endoluminal’:ab,ti,kw OR ’endoluminal surgery’:ab,ti,kw OR ’endoscopic sleeve’:ab,ti,kw OR ’endoscopic gastroplasty’:ab,ti,kw OR ’endoscopic gastric plication’:ab,ti,kw OR ’overstitch’:ab,ti,kw OR ’Apollo’:ab,ti,kw OR ’AspireAssist’:ab,ti,kw OR ’aspiration therapy’:ab,ti,kw OR ’gastrostomy tube’:ab,ti,kw OR ’botulinum’:ab,ti,kw OR ’botox’:ab,ti,kw OR ’BTX-A’:ab,ti,kw OR ’gastrostomy tube’:ab,ti,kw #3 #1 AND #2 #4 #3 AND ([article]/lim OR [article in press]/lim OR [review]/lim) AND [English]/lim #5 #4 AND ’randomized controlled trial’/de
**Database: Cochrane Library**
#1 ’obesity’:ab,ti,kw #2 MeSH descriptor: [obesity] explode all trees #3 ’endoscopic bariatric therapy’:ab,ti,kw OR ’bariatric endoscopy’:ab,ti,kw OR ’intragastric balloon’:ab,ti,kw OR ’gastric balloon’:ab,ti,kw OR ’stomach balloon’:ab,ti,kw OR ’stomach bubbles’:ab,ti,kw OR ’Ballobes’:ab,ti,kw OR ’BioEnterics’:ab,ti,kw OR ’Reshape’:ab,ti,kw OR ’BIB’:ab,ti,kw OR ’Obalon’:ab,ti,kw OR ’Obera’:ab,ti,kw OR ’Heliosphere’:ab,ti,kw OR ’Garren-Edwards’:ab,ti,kw OR ’Taylor’:ab,ti,kw OR ’Endobarrier’:ab,ti,kw OR ’Liner’:ab,ti,kw OR ’Duodeno-jenjunal bypass’:ab,ti,kw OR ’Duodenojenjunal bypass’:ab,ti,kw OR ’POSE’:ab,ti,kw OR ’primary obesity surgery endoluminal’:ab,ti,kw OR ’endoluminal surgery’:ab,ti,kw OR ’endoscopic sleeve’:ab,ti,kw OR ’endoscopic gastroplasty’:ab,ti,kw OR ’endoscopic gastric plication’:ab,ti,kw OR ’overstitch’:ab,ti,kw OR ’Apollo’:ab,ti,kw OR ’AspireAssist’:ab,ti,kw OR ’aspiration therapy’:ab,ti,kw OR ’gastrostomy tube’:ab,ti,kw OR ’botulinum’:ab,ti,kw OR ’botox’:ab,ti,kw OR ’BTX-A’:ab,ti,kw OR ’gastrostomy tube’:ab,ti,kw #4 #1 or #2 #4 #3 and #4 #5 #5 and ’random’:ab,ti,kw

### Assessment of methodological quality

The methodological quality of the finally included articles will be assessed by two authors (C.S.B., and J.J.L) using the Cochrane Risk of Bias assessment tool version 2.0 (ROB 2.0) [15]. This tool comprises five domains, including bias arising from the randomization process, bias due to deviations from intended interventions, bias due to missing outcome data, bias in measurement of the outcome and bias in selection of the reported result. Each domain comprises a series of signalling questions. Once the signalling questions are answered, the next step is to reach a risk-of-bias judgement, and assign one of three levels to each domain: Low risk of bias, Some concerns or High risk of bias [15].

The overall assessment standard of methodological quality in each study is as follows; Overall low risk of bias is judged to be at low risk of bias for all domains for this result. Overall some concerns is judged to raise some concerns in at least one domain for this result, but not to be at high risk of bias for any domain. Overall high risk of bias is judged to be at high risk of bias in at least one domain for this result or to have some concerns for multiple domains in a way that substantially lowers confidence in the result [15].

### Data extraction and additional analyses

Two authors (C.S.B. and E.J.G.) will independently extract the data from each included study and will cross-check the collected data. In cases where data are unclear, the corresponding author of the study will be contacted by e-mail to obtain insight into the original dataset. A descriptive synthesis will be developed by a systematic review process, and network meta-analysis will be performed if the included studies are sufficiently homogenous.

For additional analysis, such as subgroup analysis or meta-regression, the authors will extract the other relevant variables from each included study, such as the age, sex of the participants, geographic origin of the data, year of publication, number of total included patients, type of EBTs, and follow-up duration, etc.

### Statistics

Among the statistical approaches of network meta-analysis, frequentist frameworks will be used for this study [16]. Although the Bayesian analysis has been used for the network meta-analysis [17], this poses many limitations, such as the problem of establishing prior probability is rather more complex than the problem of testing the research hypothesis [16]. The following approach is the framework of the frequentist network meta-analysis using random-effects models and we will perform statistical analysis in the following order; 1. generating network geometry to provide an overview of the network relationship. 2 testing for inconsistency (checks the assumption of consistency). 3. creating plots and league table of effect size by treatment in order to illustrate the summary size of comparative effectiveness among various interventions. 4.: determining relative rankings of treatments (cumulative rankings for identifying superiority among interventions). 5. checking for publication bias or effect modifiers for a valid inference from results [16]. STATA software version 15.1 (College Station, Texas, US), including the packages ‘network’ and ‘mvmeta’, will be used for the network meta-analysis.

### Ethics

Approval from the institutional review board of the Chuncheon Sacred Heart Hospital was exempted because only anonymized data will be collected from the publications included in the review.

## Discussion

This updated systematic review and network meta-analysis will provide information about comparative efficacy of various EBTs and this will help physicians in the decision-making process for the selection of treatment modalities in the clinical practice.

### Expected benefits

The previously published meta-analyses were different in terms of the type of EBTs enrolled and the format of the included studies [8, 9]. Both studies were conducted 4–5 years ago and the evidence needs to be updated. This study will provide updated information to clinicians treating obesity and evaluate the quality of the synthesized evidence to help guide treatment decisions. It will also find reasons for heterogeneity among studies that were lacking in previous studies and provide future research perspectives for relevant researchers.

### Limitations

If the number of relevant studies conducted in the last 4–5 years is small, it is likely that the synthesized evidence has not been updated much. However, we will follow the protocol of this study to address the limitations of previous studies.

## Supporting information

S1 Checklist PRISMA-P (Preferred Reporting Items for Systematic Review and Meta-Analysis Protocols) 2015 checklist: Recommended items to address in a systematic review protocol. The PRISMA-P 2015 checklist for items to address in a systematic review protocol.
(DOC)

## Abbreviations

EBTs: endoscopic bariatric treatments
RCTs: randomized controlled trials
%EWL: percent excess weight loss
%TBWL: percent total body weight loss
ESG: endoscopic sleeve gastroplasty
DJBL: duodenal-jejunal bypass liner
AA: AspireAssist
POSE: primary obesity surgery endoluminal
